# Effect of Different Doses of Aerobic Exercise on Total White Blood Cell (WBC) and WBC Subfraction Number in Postmenopausal Women: Results from DREW

**DOI:** 10.1371/journal.pone.0031319

**Published:** 2012-02-17

**Authors:** Neil M. Johannsen, Damon L. Swift, William D. Johnson, Vishwa D. Dixit, Conrad P. Earnest, Steven N. Blair, Timothy S. Church

**Affiliations:** 1 Department of Preventive Medicine, Pennington Biomedical Research Center, Baton Rouge, Louisiana, United States of America; 2 Department of Biostatistics, Pennington Biomedical Research Center, Baton Rouge, Louisiana, United States of America; 3 Immunobiology Laboratory, Pennington Biomedical Research Center, Baton Rouge, Louisiana, United States of America; 4 Department of Exercise Science and Department of Epidemiology and Biostatistics, University of South Carolina, Columbia, South Carolina, United States of America; University of Las Palmas de Gran Canaria, Spain

## Abstract

**Background:**

Elevated total white blood cell (WBC) count is associated with an increased risk of coronary heart disease and death. Aerobic exercise is associated with lower total WBC, neutrophil, and monocyte counts. However, no studies have evaluated the effect of the amount of aerobic exercise (dose) on total WBC and WBC subfraction counts.

**Purpose:**

To examine the effects of 3 different doses of aerobic exercise on changes in total WBC and WBC subfraction counts and independent effects of changes in fitness, adiposity, markers of inflammation (IL-6, TNF-α, C-reactive protein), fasting glucose metabolism, and adiponectin.

**Methods:**

Data from 390 sedentary, overweight/obese postmenopausal women from the DREW study were used in these analyses. Women were randomized to a non-exercise control group or one of 3 exercise groups: energy expenditure of 4, 8, or 12 kcal kg^−1^⋅week^−1^ (KKW) for 6 months at an intensity of 50% VO_2peak_.

**Results:**

A dose-dependent decrease in total WBC counts (trend *P* = 0.002) was observed with a significant decrease in the 12KKW group (−163.1±140.0 cells/µL; mean±95%CI) compared with the control (138.6±144.7 cells/µL). A similar response was seen in the neutrophil subfraction (trend *P* = 0.001) with a significant decrease in the 12KKW group (−152.6±115.1 cells/µL) compared with both the control and 4KKW groups (96.4±119.0 and 21.9±95.3 cells/µL, respectively) and in the 8KKW group (−102.4±125.0 cells/µL) compared with the control. When divided into high/low baseline WBC categories (median split), a dose-dependent decrease in both total WBCs (P = 0.003) and neutrophils (P<0.001) was observed in women with high baseline WBC counts. The effects of exercise dose on total WBC and neutrophil counts persisted after accounting for significant independent effects of change in waist circumference and IL-6.

**Conclusion:**

Aerobic exercise training reduces total WBC and neutrophil counts, in a dose-dependent manner, in overweight/obese postmenopausal women and is especially beneficial for those with systemic low grade inflammation.

**Clinical Trials Identifier: NCT00011193:**

## Introduction

Elevated white blood cell (WBC) count is a strong independent risk factor for coronary heart disease (CHD) morbidity and mortality [1]–[4]. Epidemiologic studies suggest that greater total granulocyte or neutrophil counts are the strongest predictor [5]–[8] accounting for an increased risk of CVD death of approximately 40% [5]. Total WBC count is also associated with insulin sensitivity such that an increase in total WBC count is indicative of an increased risk of future type 2 diabetes mellitus [9], [10]. Recently, Dixon and O'Brien [11] demonstrated that total WBC, and especially the neutrophil subclass, were associated with BMI and independently associated with insulin concentrations. Postmenopausal women represent a unique demographic deserving investigation because they have additional risk factors including elevated systolic blood pressure, deteriorating blood lipid profile, increasing body weight and low levels of physical activity that, together with elevated WBC counts, could result in a heightened CVD and type 2 diabetes risk [10], [12], [13].

Recently, we reported that fitness (inversely) and fatness (directly) are associated with total and fractionated WBC counts [14], [15]. Men with low fitness and high fatness had higher total WBC, neutrophil, lymphocyte, and basophil counts compared to men with high fitness levels. While acute exercise bouts have been implicated in an augmented inflammatory state [16], high levels of physical activity have been linked to reduced systemic inflammation and aerobic exercise training has been shown to decrease WBC counts [17] and associated inflammatory biomarkers (ex. IL-6) [18]. However, the dose of exercise necessary to improve total and fractionated WBC counts and their relationship with exercise-induced changes in adiposity, inflammatory biomarkers, and fasting glucose metabolism in postmenopausal women has not been examined. The Dose-Response to Exercise in Women Aged 45–75 yr (DREW) study provides a unique opportunity to evaluate the dose of exercise necessary to promote improvements in total WBC and WBC subfraction counts. We hypothesized that total WBC and WBC subfraction counts would be reduced to a greater extent at higher doses of exercise. We also hypothesized that the reduction in total WBC and WBC subfraction counts would be related to improvements in cardiorespiratory fitness (VO_2peak_), and markers of adiposity (BMI and waist circumference), inflammatory cytokines and adipokines (IL-6, TNF-α, C-reactive protein, and adiponectin), and fasting glucose metabolism (glucose, insulin, and calculated HOMA).

## Methods

### Study design and participants

The DREW study was approved annually by The Cooper Institute during data collection and subsequently by the Pennington Biomedical Research Center institutional review board for continued analyses. Written informed consent was obtained from all participants prior to study screening. The design and methods for the DREW study and the primary outcomes have been previously published [19], [20]. Briefly, DREW was a randomized, controlled intervention designed to examine the effect of aerobic exercise dose on improvements in cardiorespiratory fitness in 464 women aged 45 to 75 years. Women were randomized into either a non-exercise control group or one of 3 exercise intervention groups with incrementally higher doses of energy expenditure. The participants in this study were sedentary (aerobic exercise<20 min on <3 d/wk and taking <8000 steps/d), overweight and obese (BMI 25.0–43.0 kg/m^2^), postmenopausal women with elevated systolic blood pressure (range 120.0–159.9 mmHg). Women were excluded from participation if they had a past history of significant cardiovascular disease, elevated low-density lipoproteins (≥130 mg/dL), or other medical condition that would interfere with or be aggravated by exercise. A comprehensive description of the recruiting, screening, and study procedure has been published [19].

### Intervention

Exercise was prescribed in increments according to consensus public health recommendations set forth by the NIH and others [21]–[23]. We previously calculated the average energy expenditure required for sedentary women in this age group to achieve the recommended dose of exercise to be 8 kcal kg^−1^ week^−1^ (KKW). The primary aim of the study was to evaluate whether minimal (50% of recommended dose) or optimal (150% of recommended dose) levels of exercise exist for improvements in fitness as measured by change in VO_2peak_. Thus, the exercise doses utilized in DREW were 4, 8, and 12 KKW. Women randomized to an exercise group participated in 3 or 4 training sessions per week for 6 months at an intensity equivalent to the heart rate associated with 50% VO_2peak_. The exercise intervention consisted of alternating sessions of treadmill walking and semi-recumbent cycle ergometry. All exercise groups completed 4 KKW in the first week of exercise. Women randomized to the 4 KKW group completed this dose for the entire 6 month intervention period. Women randomized to the 8 KKW and 12 KKW groups increased their dose of exercise by 1 KKW per week until the appropriate dose was achieved. All exercise sessions were monitored by trained staff to assure the appropriate exercise dose was achieved and participant safety was maintained during the intervention. Exercising energy expenditure rate (kcal/min) was calculated using weekly body weight and either treadmill speed and grade or cycle ergometry Watts and standard American College of Sports Medicine (ACSM) equations [24]. Session exercise time was calculated by dividing required daily caloric dose (weekly kcal/number of sessions) by estimated energy expenditure rate. Adherence rates (total exercise kcal expenditure/total prescribed kcals ×100) for the exercise groups were very high and ranged from 89.0% (8 KKW) to 94.6% (4 KKW) [20].

Women randomized into the non-exercise control group were asked to maintain their current level of physical activity throughout the entire 6-month study period. Caloric intake and nutritional composition of the participant's diet were not controlled during the intervention. Separate intervention and clinical assessment teams were used to assure the assessment team was blinded to randomization. Participants were reminded regularly not to discuss their randomization with assessment team members.

### Clinical Measures

Age, ethnicity, smoking history and alcohol use were obtained by self-report during baseline and follow-up assessment periods. At baseline and follow-up, participants completed a medical history form and medication inventory and had a basic exam conducted by a physician. Weight was measured on an electronic scale (Siemens Medical Solutions, Malvern, PA) and height was measured on a wall stadiometer. Body mass index (BMI) was calculated as body weight in kg divided by height in meters squared. Peak oxygen consumption (VO_2peak_) was measured twice at baseline and twice at follow-up on two separate days on an electronically-braked cycle ergometer (Lode, Groningen, The Netherlands) and is reported as the mean of the duplicate baseline and follow-up measurements. The maximal graded exercise test consisted of participants cycling for 2 minutes at 30 Watts, 4 minutes at 50 Watts, then increments of 20 Watts every 2 minutes until pedal cadence could not be maintained above 50 RPM. Heart rate was monitored by 12 lead ECG and respiratory gasses (VO_2_ and VCO_2_) were analyzed using a metabolic cart (Parvomedics True Max 2400, Salt Lake City, UT) calibrated with standard gas mixtures. Exact methodology and reproducibility (intraclass correlations) have been reported in previous manuscripts [19], [20].

Hematology was conducted by antecubital venipuncture at baseline and after 6 months of intervention with blood collection occurring within 2–3 days of their final exercise session. Blood samples were analyzed for routine CBC including total WBC count and differential for WBC subfractions (Pentra 60, ABX Diagnostics, France). The reported linearity limits for total WBC are 0 to 120×10^−3^ cells/µL with a CV<2.0%. The quantity of each WBC subfraction (lymphocytes, monocytes, neutrophils, basophils, and eosinophils; cells/µL) was calculated by multiplying the measured fraction of each by the total WBC count. Fasting plasma glucose was measured enzymatically by the hexokinase-glucose-6-phophate dehydrogenase method and insulin was measured by electrochemiluminescence. Homeostatic model assessment for insulin resistance (HOMA) was calculated as a surrogate marker for determining impaired fasting glucose metabolism [25]. Plasma adiponectin (B-Bridge International, Inc., San Jose, CA) and IL-6 and TNF-α (R&D Systems, Inc., Minneapolis, MN) were measured by ELISA. High sensitivity C-reactive protein (CRP) was measured on a Prospect nephelometer (Dade Division of Baxter Healthcare Corporation, Delaware, MD). The Cooper Clinic laboratory participates in and meets the quality control standards of the CLIA (Clinical Laboratory Information Act).

### Statistical Analyses

All data were analyzed using JMP 9.0.2 (SAS® Institute Inc. Cary, NC) statistical software. Participants with incomplete data were not used in the final analysis. One-way analysis of variance (ANOVA) was used to assess significance of group differences at baseline. Spearman correlations (*r_S_*) were used to examine the relationships between changes (follow-up minus baseline) in anthropometric data and cardiorespiratory fitness (VO_2peak_) and total WBC and WBC subfraction counts after 6-months of intervention. Analysis of covariance (ANCOVA) was used to determine the effect of intervention on the change in anthropometric data, fitness, and leukocyte counts after adjusting for respective baseline assessments. We further investigated whether changes in fitness, BMI and waist circumference mediated the effects of exercise dose on baseline adjusted-total WBC and WBC subfraction counts. We also determined whether participants with the highest total WBC and neutrophil counts, and subsequently at greatest risk of CHD [8], had the greatest reductions in WBC counts in response to exercise. For this analysis, total WBC and the neutrophil fraction were classified by median split of baseline values into high and low categories and change in total WBCs and neutrophils were compared across intervention groups within strata.

A secondary analysis of the above responses was conducted using only participants with complete data for inflammatory cytokines (IL-6, TNF-α, and CRP), adiponectin, and markers of glucose regulation (fasting glucose, insulin, and HOMA). Initially, baseline-adjusted changes in cytokines, adiponectin and markers of glucose regulation were analyzed across intervention groups and Spearman correlations (r_S_) against total WBC and each WBC subfraction were used to elucidate potential covariates. Significant covariates were entered into baseline-adjusted ANCOVAs for change in total WBC and WBC subfractions and changes in WBCs by baseline WBC median split. Post-hoc analyses for within and between group differences in both the primary and secondary analyses were conducted using the least significant difference (LSD) method for multiple comparisons and further follow-up analysis was done on each high/low category separately to test for a general linear trend across intervention groups. All data are summarized as mean±95%CI unless stated otherwise and statistical significance was set at *P*<0.05.

## Results

**Figure 1:**
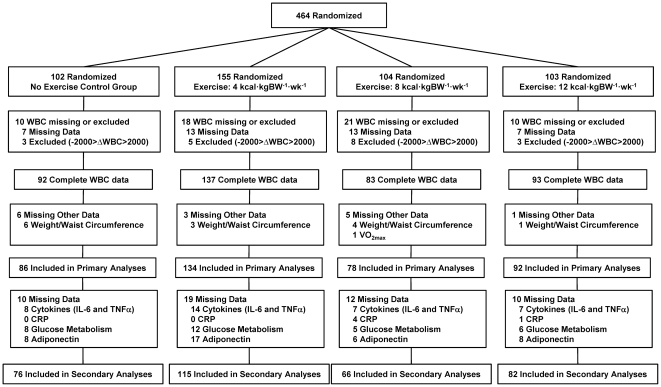
CONSORT schematic of study participation.

Study specific reasons for excluding participant data are shown in Figure 1. For the primary analyses, 409 participants had complete WBC and anthropometric data at baseline and after 6 months of intervention. Additionally, we removed 19 outliers from the main analyses with a large increase (ΔWBC>2000) or decrease (ΔWBC<−2000) in total WBC counts. The outliers were distributed across all groups (n = 3, 5, 8, and 3 for the Control, 4, 8, and 12 KKW groups, respectively) and similar results were observed when all participants with complete WBC data were included in the analysis (data not shown). The secondary analyses included 339 participants with complete baseline and follow-up inflammation (IL-6, TNF-α, and CRP), glucose metabolism (fasting glucose, insulin and calculated HOMA), and adiponectin data. The breakdown of missing data from the primary analyses is also located in Figure 1. The results for the primary analyses will be reported initially followed by the secondary analyses. There were no significant differences in the results for the primary outcomes after removing the participants with incomplete data for the secondary analyses (data not shown).

Baseline demographic and anthropometric data, VO_2peak_, and unadjusted total WBC count and WBC subfraction concentrations are presented in Table 1. Mean (±SD) age, BMI, and waist circumference, were 57.3±6.4 y, 31.7±3.8 kg/m^2^, and 100.8±11.5 cm, respectively. The study sample was an ethnically diverse (36.9% non-Caucasian) group of postmenopausal women with low baseline fitness levels (VO_2peak_ = 15.5±2.9 ml kg^−1^ min^−1^). No significant differences were observed among intervention groups for baseline data (*P*>0.10).

**Table 1:** Baseline participant characteristics and unadjusted leukocyte data.

			Treatment Group							
	All		Control		4 KKW		8 KKW		12 KKW	
n	390		86		134		78		92	
Age, y	57.3	(6.4)	57.1	(5.7)	58.1	(6.6)	57.2	(6.8)	56.6	(6.5)
Ethnicity, n (%)										
White	246	(63.1)	53	(61.6)	80	(59.7)	48	(61.5)	65	(70.7)
African American	114	(29.2)	23	(26.7)	45	(33.6)	23	(29.5)	23	(25.0)
Other	30	(7.7)	10	(11.6)	9	(6.7)	7	(9.0)	4	(4.4)
Body Weight, kg	84.2	(11.7)	85.4	(12.2)	83.5	(11.3)	84.9	(12.4)	83.6	(11.2)
BMI, kg/m^2^	31.7	(3.8)	32.1	(3.9)	31.5	(3.7)	32.2	(4.1)	31.2	(3.5)
Waist Circumference, cm	100.8	(11.5)	102.2	(11.4)	100.1	(11.2)	101.9	(11.6)	99.7	(11.9)
VO_2peak_, mL⋅kg^−1^⋅min^−1^	15.5	(2.9)	15.7	(2.9)	15.4	(3.0)	14.9	(2.4)	15.9	(2.9)
Total WBC, cells/ìL	5662	(1539)	5619	(1588)	5718	(1391)	5776	(1594)	5526	(1658)
Lymphocytes, cells/µL	1926	(558)	1901	(494)	1962	(577)	1976	(605)	1854	(547)
Monocytes, cells/µL	383	(123)	376	(118)	386	(115)	368	(111)	396	(148)
Neutrophils, cells/µL	3144	(1158)	3128	(1145)	3151	(1078)	3230	(1044)	3074	(1369)
Basophils, cells/µL	34.9	(19.6)	35.3	(17.9)	34.5	(14.3)	37.9	(30.4)	32.8	(15.6)
Eosinophils, cells/µL	175	(126)	179	(146)	185	(146)	164	(88)	169	(99)

Data are presented as mean (SD). BMI, body mass index calculated as weight in kg divided by height in meters squared; VO_2_, volume of oxygen consumed; WBC, white blood cell; KKW, kcal per kg body weight per week.

Baseline adjusted changes in anthropometric and fitness (VO_2peak_) data and total WBC and WBC subfraction counts after 6 months of intervention are shown in Table 2. Similar changes in fitness and waist circumference were observed in this cohort compared with the entire DREW sample [20]. Change in BMI was not significantly different across the intervention groups; however, all groups experienced a decrease in BMI after the intervention (Table 2). Change in total WBC counts was dose dependent (trend *P* = 0.002) with a significant reduction after 12 KKW compared with control (*P*<0.05). Change in neutrophil counts was similar to total WBCs (trend *P* = 0.001) with a significant reduction after 8 KKW and 12 KKW compared to control and after 12 KKW compared to 4 KKW (*P*<0.05). Lymphocyte, monocyte, basophil and eosinophil counts did not change significantly across exercise doses.

**Table 2:** Mean (95%CI) change in anthropometric and fitness data and total WBC and WBC subfraction counts after 6 months of intervention.

	Control	4 KKW	8 KKW	12 KKW	*P*-value (trend)
Body Weight, kg	−1.11	−1.15	−1.80	−1.36	*0.50*
	(−1.80, −0.41)	(−1.71, −0.60)	(−2.52, −1.07)	(−2.03, −0.69)	*(0.40)*
BMI, kg/m^2^	−0.46	−0.45	−0.72	−0.56	*0.43*
	(−0.72, −0.19)	(−0.66, −0.24)	(−1.00, −0.45)	(−0.82, −0.31)	*(0.31)*
Waist Circumference, cm	0.50	−2.67*	−2.47*	−2.85*	*<0.003*
	(−0.96, 1.96)	(−3.83, −1.50)	(−4.00, −0.94)	(−4.26, −1.44)	*(0.005)*
VO_2peak_, mL⋅kg^−1^⋅min^−1^	−0.16	0.57*	1.28* †	1.62* †	*<0.001*
	(−0.53, 0.21)	(0.27, 0.86)	(0.89, 1.67)	(1.27, 1.98)	*(<0.001)*
Total WBC, cells/µL	138.6	−14.5	−116.3	−163.1*	<0.02
	(−6.1, 283.3)	(−130.5, 101.4)	(−268.3, 35.7)	(−303.1, −23.0)	(0.002)
Lymphocytes, cells/µL	21.6	−30.0	−18.7	−16.0	0.65
	(−41.7, 84.9)	(−80.8, 20.7)	(−85.3, 47.8)	(−77.4, 45.3)	(0.53)
Monocytes, cells/µL	12.8	−2.3	−6.7	0.0	0.45
	(−4.7, 30.3)	(−16.4, 11.7)	(−25.1, 11.7)	(−16.9, 17.0)	(0.32)
Neutrophils, cells/µL	96.4	21.9	−102.4*	−152.6* †	0.01
	(−22.6, 215.4)	(−73.5, 117.2)	(−227.4, 22.6)	(−267.7, −37.5)	(0.001)
Basophils, cells/µL	3.7	3.3	0.0	0.7	0.41
	(−0.1, 7.4)	(0.3, 6.3)	(−4.0, 4.0)	(−2.9, 4.4)	(0.14)
Eosinophils, cells/µL	1.6	−4.0	13.5	−0.1	0.46
	(−14.7, 17.9)	(−17.0, 9.1)	(−3.6, 30.6)	(−15.8, 15.7)	(0.71)

Data are presented as mean (95%CI). *P*-values are from ANCOVA after adjusting for baseline values and using general linear models for trends. WBC, white blood cell; KKW, kcal per kg body weight per week;

*P<0.05 v. Control;

† *P*<0.05 v. 4 KKW.

Table 3 demonstrates the associations between the change in total WBC and WBC subfraction counts and change in anthropometrics and cardiorespiratory fitness. Change in waist circumference was positively correlated with change in total WBC (*r_S_* = 0.13, *P* = 0.008) as well as lymphocyte (*r_S_* = 0.18, *P*<0.001), monocyte (*r_S_* = 0.16, *P* = 0.002), and basophil (*r_S_* = 0.11, *P*<0.03) counts. Age was inversely associated with change in neutrophil counts (*r_S_* = −0.10, *P* = 0.04). Changes in fitness, body weight, and BMI after the intervention were not associated with change in total WBC or WBC subfraction counts.

**Table 3:** Spearman correlation coefficients (r_S_) between change in total WBC and WBC subfraction counts (cells/µL) and changes in anthropometric data and cardiorespiratory fitness after 6 months of intervention.

	Total WBC		Lymphocytes		Monocytes		Neutrophils		Basophils		Eosinophils	
	*r_S_*	*P*	*r_S_*	*P*	*r_S_*	*P*	*r_S_*	*P*	*r_S_*	*P*	*r_S_*	*P*
Age, y	−0.05	0.31	0.04	0.42	−0.01	0.86	**−0.10**	**0.04**	−0.03	0.51	0.01	0.89
Body Weight, kg	0.00	0.98	0.06	0.23	0.01	0.77	−0.02	0.68	0.01	0.88	−0.09	0.08
BMI, kg/m^2^	0.01	0.79	0.07	0.16	0.02	0.71	−0.01	0.85	0.02	0.66	−0.08	0.11
Waist Circumference, cm	**0.13**	**0.008**	**0.18**	**<0.001**	**0.16**	**0.002**	0.06	0.24	**0.11**	**<0.03**	−0.01	0.92
VO_2peak_, mL⋅kg^−1^⋅min^−1^	−0.03	0.63	0.01	0.88	−0.02	0.67	−0.03	0.50	−0.01	0.88	0.09	0.07

Data are presented as Spearman's rho (*r_S_*) and *P*-values from simple linear correlations. WBC, white blood cell; BMI, body mass index calculated as weight in kg divided by height in meters squared; VO_2_ volume of oxygen consumed. Bolded values are statistically significant at *P*<0.05.

Changes in waist circumference, BMI and fitness were entered separately and together into the statistical models in Table 2 to investigate the independent effect of each on changes in total WBC and WBC subfraction counts. Change in waist circumference (semi-partial r = 0.17, *P*<0.001) and exercise dose (trend *P* = 0.007) remained significant independent predictors of change in total WBC count. This result was the combined effect of the positive association of change in waist circumference on lymphocyte, monocyte, and basophil counts (Table 3) and inverse effect exercise dose on neutrophil count (Table 2).

**Figure 2:**
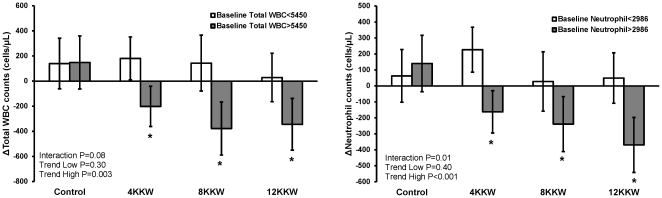
Change (Δ) in total WBC and neutrophil counts by exercise group (control, 4, 8, and 12 kcal kg^−1^ min^−1^; KKW) and high/low baseline WBC counts. Interaction  =  group x high/low WBC category; trend high and low are from general linear model across intervention groups separately for each high/low baseline WBC category. The participant numbers (n) in the high baseline total WBC groups were 41, 71, 41, and 43 and low baseline total WBC groups were 45, 63, 37, and 49 for Control, 4, 8, and 12 KKW, respectively. The participant numbers (n) in the high baseline neutrophil groups were 40, 71, 42, and 42 and low baseline neutrophil groups were 46, 63, 36, and 50 for Control, 4, 8, and 12 KKW, respectively. * Significantly different between high/low baseline WBC categories within an exercise group.

Change in total WBC and neutrophil counts, categorized by baseline WBC level (median split) and intervention group, are shown in Figure 2. The interaction between high/low baseline WBC category and intervention group was significant for change neutrophils (*P* = 0.01) and approached significance for change in total WBCs (*P* = 0.08). Follow-up trend analyses suggested that women with the highest baseline total WBC counts had the largest reductions in total WBC (trend *P* = 0.003) and neutrophil counts (trend *P*<0.001). Exercising women with high total WBC and neutrophil counts also had a significant reduction in total WBC and neutrophils (*P*<0.05 for all) compared to control women and women with low WBC counts exercising at a similar dose. No significant changes in total WBC and neutrophil counts were observed in women with low baseline WBC counts (Figure 2).

Table 4 shows the results of the secondary analyses for dose dependent changes in markers of fasting glucose metabolism, inflammation and adiponectin after 6 months of intervention. In this cohort, fasting glucose was significantly lower after exercise (trend *P* = 0.006) compared with Control due to lower fasting glucose levels in all exercise groups (*P*<0.05). No other indicators of fasting glucose metabolism or inflammation responded differently dependent on exercise dose.

**Table 4:** Mean (95%CI) change in markers of fasting glucose metabolism and inflammation after 6 months of intervention.

	Control	4 KKW	8 KKW	12 KKW	*P*-value (trend)
Glucose, mg/dL	1.21	−1.21*	−1.34*	−2.09*	**0.02**
	(−0.37, 2.79)	(−2.49, 0.07)	(−3.04, 0.35)	(−3.61, −0.57)	**(0.006)**
Insulin, pmol/L	0.63	−0.53	−5.19	−2.76	0.59
	(−5.59, 6.85)	(−5.59, 4.52)	(−11.86, 1.48)	(−8.75, 3.23)	(0.29)
HOMA	0.05	−0.08	−0.23	−0.15	0.56
	(−0.22, 0.32)	(−0.30, 0.14)	(−0.52, 0.06)	(−0.41, 0.11)	(0.24)
Adiponectin, µg/mL	−0.12	−0.02	−0.00	−0.26	0.69
	(−0.47, 0.23)	(−0.30, 0.27)	(−0.37, 0.37)	(−0.60, 0.08)	(0.54)
IL-6, pg/mL	−0.00	0.01	0.16	−0.20	0.32
	(−0.27, 0.27)	(−0.21, 0.23)	(−0.13, 0.46)	(−0.46, 0.06)	(0.40)
TNF-α, pg/mL	−0.09	0.01	0.19	0.08	0.14
	(−0.26, 0.07)	(−0.12, 0.15)	(0.01, 0.37)	(−0.08, 0.24)	(0.08)
CRP, mg/L	−0.35	0.03	−0.45	0.05	0.77
	(−1.19, 0.49)	(−0.66, 0.71)	(−1.36, 0.45)	(−0.76, 0.86)	(0.72)

Data are presented as mean (95%CI) in participants with complete data for WBC counts, fitness, and markers of adiposity, fasting glucose metabolism, and inflammation (n = 339). *P*-values are from ANCOVA after adjusting for baseline values and using general linear models for trends. KKW, kcal per kg body weight per week; HOMA, homeostasis model assessment of insulin resistance; IL-6, interleukin-6; TNF-α, tumor necrosis factor-α; CRP, high sensitivity C-reactive protein;

*P<0.05 v. Control.

Table 5 shows the secondary analyses for associations between the change in total WBC and WBC subfractions and changes in markers of fasting glucose metabolism, inflammation and adiponectin. The change in the inflammatory cytokine IL-6 was positively associated with change in total WBC count (r_S_ = 0.26, *P*<0.001) as well as changes in the monocyte (r_S_ = 0.23, *P*<0.001) and neutrophil (r_S_ = 0.25, *P*<0.001) subfractions. The change in CRP was positively correlated with the change in monocyte (r_S_ = 0.12, *P*<0.03), neutrophil (r_S_ = 0.11, *P*<0.05), and basophil (r_S_ = 0.11, *P*<0.05) counts and tended to be positively associated with the change in total WBC count (r_S_ = 0.10, *P*<0.06). The change in fasting glucose level was directly associated with the change in monocyte count (r_S_ = 0.11, *P* = 0.04) and the change in adiponectin was negatively associated with the change in lymphocyte count (r_S_ = −0.15, *P*<0.006). No other changes in fasting glucose metabolism or inflammation were associated with changes in WBC populations.

**Table 5:** Spearman correlation coefficients (r_S_) between change in total WBC and WBC subfraction counts (cells/µL) and changes in fasting glucose metabolism and inflammation after 6 months of intervention.

	Total WBC		Lymphocytes		Monocytes		Neutrophils		Basophils		Eosinophils	
	*r_S_*	*P*	*r_S_*	*P*	*r_S_*	*P*	*r_S_*	*P*	*r_S_*	*P*	*r_S_*	*P*
Glucose, mg/dL	0.03	0.58	−0.09	<0.10	**0.11**	**0.04**	0.06	0.29	−0.05	0.32	0.00	0.98
Insulin, pmol/L	0.06	0.24	0.00	0.98	0.09	0.08	0.05	0.32	0.02	0.65	−0.02	0.69
HOMA	0.06	0.25	−0.01	0.92	0.10	<0.06	0.05	0.32	0.02	0.70	−0.00	0.94
Adiponectin, µg/mL	−0.02	0.71	**−0.15**	**<0.006**	−0.03	0.57	0.06	0.28	0.05	0.40	−0.05	0.32
IL-6, pg/mL	**0.26**	**<0.001**	0.04	0.42	**0.23**	**<0.001**	**0.25**	**<0.001**	0.10	0.07	0.10	<0.07
TNF-α, pg/mL	−0.04	0.46	−0.02	0.70	0.05	0.41	−0.06	0.27	0.04	0.51	0.06	0.27
CRP, mg/L	0.10	<0.06	−0.06	0.28	**0.12**	**<0.03**	**0.11**	**<0.05**	**0.11**	**<0.05**	−0.00	0.94

Data are presented as Spearman's rho (*r_S_*) and *P*-values from simple linear correlations from participants with complete data for WBC counts, fitness, and markers of adiposity, fasting glucose metabolism, and inflammation (n = 339). HOMA, homeostasis model assessment of insulin resistance; IL-6, interleukin-6; TNF-α, tumor necrosis factor-α; CRP, high sensitivity C-reactive protein. Bolded values are statistically significant at *P*<0.05.

Also in our secondary analyses, we determined the independent effects of exercise dose and changes in markers of adiposity, fitness, fasting glucose metabolism, inflammation, and adiponectin on total WBC and WBC subfractions. The baseline-adjusted decrease in total WBC counts was dose dependent (trend *P* = 0.003) with the greatest decrease in the 12 KKW group (−191±143 cell/µL) compared with both the control and 4 KKW groups (111±149 and −2±120 cells/µL, respectively; *P*<0.05) even after accounting for the independent effects of change in waist circumference (semi-partial r = 0.09; *P* = 0.08) and change in IL-6 (semi-partial r = 0.20; *P*<0.001). After adjusting for age (semi-partial r = 0.09, *P* = 0.07) and change in IL-6 (semi-partial r = 0.23; *P*<0.001), the baseline-adjusted change in neutrophil count was also dose dependent (trend *P*<0.001) with the greatest decrease after the 12 KKW group (194±119 cells/µL) compared with both the control and 4 KKW groups (73±123 and 39±100 cells/µL, respectively; *P*<0.05). Neither change in CRP (*P* = 0.64) nor change in waist circumference (*P* = 0.80) had further influence on the change in neutrophil count. Furthermore, the changes in total WBC and neutrophil counts were not associated with a change in fitness (VO_2peak_).

Although intervention group did not influence monocyte, lymphocyte, basophil, or eosinophil counts, we did exploratory analyses to determine factors responsible for the post-intervention changes after adjusting for their respective baseline values and intervention group. The change in monocyte count was positively associated with change in waist circumference (semi-partial r = 0.11; *P* = 0.03) and change in IL-6 (semi-partial r = 0.18; *P*<0.001). The change in lymphocyte count after the intervention was negatively associated with change in adiponectin levels (semi-partial r = −0.10; *P*<0.04) and positively associated with change in waist circumference (semi-partial r = 0.16; *P*<0.002) and change in IL-6 (semi-partial r = 0.10; *P* = 0.04). No significant baseline and intervention group adjusted changes in basophil or eosinophil counts were associated with any markers of adiposity, fitness, glucose metabolism, inflammation, or adiponectin.

The final analyses included the effect of exercise dose and potential mediating effects of markers of adiposity, fitness, fasting glucose metabolism, inflammation, and adiponectin on total WBC and neutrophil counts after median split into high and low baseline total WBC groups. Similar results were observed in these follow-up analyses as previously demonstrated in Figure 2. Women categorized as having high baseline counts had the largest decrease in total WBC (trend *P* = 0.005) counts even after adjusting for change in waist circumference (semi-partial r = 0.09; *P*<0.09) and change in IL-6 (semi-partial r = 0.23; *P*<0.001). Similarly, neutrophil counts in exercising women with high baseline counts had a dose dependent decrease (trend *P*<0.001) even after adjusting for age (semi-partial r = −0.08; *P* = 0.12) and change in IL-6 (semi-partial r = 0.26; *P*<0.001). No significant trends were observed for total WBC (*P* = 0.24) or neutrophil (*P* = 0.21) counts in women classified into the low baseline categories.

## Discussion

The main finding from this investigation is that 6 months of aerobic exercise reduces total WBC and neutrophil counts in sedentary, overweight/obese, postmenopausal women. The data suggest that a significant reduction in total WBC and neutrophil counts can be attained with aerobic exercise levels at or above public health recommendations for improved health. Exercise, coupled with interventions specifically targeting abdominal obesity and IL-6 and adiponectin concentrations may be most effective at correcting total WBC counts by independently lowering lymphocyte, monocyte, and basophil numbers. To our knowledge, this is the first randomized trial to examine the effect of different doses of aerobic exercise on total WBC and WBC subfractions and provides further evidence that increasing physical activity levels significantly reduces factors that are related to increased risk of CVD morbidity and mortality, especially in women with systemic low grade inflammation.

Epidemiologic data suggest that higher fitness is associated with reduced total WBC counts due to lower counts in all WBC subclasses [14], [15]. Additionally, we reported that lower BMI was associated with reduced total WBC and, in particular, neutrophil, lymphocyte, monocyte, and basophil counts [14], [15]. This investigation extends the results of these epidemiologic studies by examining whether a dose dependent increase in fitness and an exercise related decrease in abdominal obesity (waist circumference) reduce total WBC and WBC subfraction counts. Increased fitness (ΔVO_2peak_) was not directly associated with improved WBC counts (Table 3) and the effects of exercise group on total WBC and neutrophil counts were independent of the change in fitness. Likewise, in our secondary analyses, we further investigated whether the observed reduction in total WBC and neutrophil counts were in response to changes in markers of inflammation, fasting glucose metabolism and adiponectin. While we did observe significant independent relationships between the changes in total WBC and neutrophil counts and change in IL-6, a significant dose-response persisted and none of the other factors included in the analyses were able to explain why increased doses of aerobic exercise training resulted in reduced WBC counts. These results suggest that changes in total WBC and neutrophil counts may be related to a dose-dependent change in unmeasured factors or an increase in physical activity behavior and not directly related to physiologic mechanisms associated with increased fitness.

Further analysis of our data revealed the greatest reduction in total WBC and neutrophil counts in women with greater CVD risk as determined by elevated baseline counts (Figure 2). Past research shows that for every increase in total WBC or granulocyte count (neutrophil+basophil+eosinophil) of 1000 cells/µL, CHD risk increases by ∼10% [8]. In the current investigation, we observed a change in total WBC count of ∼250 and 300 cells/µL in the 8 KKW and 12 KKW groups which translates respectively into a 2 and 3% reduction in CHD risk compared with the control group. Similar results were observed in the neutrophil subclass. However, when we analyzed women at the greatest risk for CHD, the risk reduction increased to 3 to 5% for total WBC and neutrophil counts across all exercise groups. These data suggest that aerobic exercise in postmenopausal women with elevated total WBC and neutrophil counts may be linked to improved CHD risk. In addition, this study suggests that total WBC and neutrophil counts could serve as potential indicators of the effectiveness of exercise regimens to correct underlying low grade inflammatory disease.

Several mechanisms exist that may explain the reduction in total WBC, and specifically neutrophil, counts with exercise training. Exercise may alter the trafficking of leukocyte subsets between secondary lymphoid organs and blood. In addition, the possibility that exercise directly impacts bone marrow hematopoiesis and thus accounts for changes in WBCs in blood warrants further investigation. Emerging evidence suggests that activation of innate and adaptive immune cell subsets causes increased inflammation in obesity [26]. A reduction in adiposity and associated restoration of adipokine balance (reduction in leptin and increased adiponectin) by exercise can contribute to changes in leukocyte trafficking, hematopoiesis or turnover of WBCs [27]. Adiposity is also highly correlated with elevated circulating leptin concentration and leptin has been shown to activate neutrophils and induce production of pro-inflammatory cytokines [28], [29]. Both TNF-α and IL-6 have been shown to decrease with long-term physical activity resulting in significant decreases in leukocytosis and neutrophilia [18]. Dixon and O'Brien [11] also demonstrated that total WBC, and especially the neutrophil subclass, were associated with BMI and independently associated with insulin concentrations. Likewise, a significant reduction in BMI after weight-loss surgery resulted in a 11.7% decrease in neutrophils and a 6.9% decrease in lymphocytes [11]. Last, total WBC count is associated with insulin sensitivity such that an increase in total WBC count is indicative of an increased risk of future type 2 diabetes mellitus [9], [10]. In our study, we observed exercise-induced reductions in waist circumference, but not BMI, were associated with a decrease in lymphocytes, monocytes, and basophils (Table 3). This is in contrast to Michishita et al. who demonstrated that percent change in BMI after 6 weeks of aerobic exercise was associated with a reduction in percent monocytes and neutrophils [17]. A recent study by Arsenault et al. from DREW data, and results from the secondary analyses in this cohort, showed no change in either TNF-α or IL-6 after 6 months of exercise([30] and Table 4). However, significant decreases in fasting glucose and adiposity levels (waist circumference) were observed across all exercise groups (Tables 2 and 4). The final analyses in this report suggest that increased physical activity can reduce total WBC and neutrophil counts independently of changes in adiposity, inflammatory cytokines (especially IL-6), and fasting glucose metabolism. Also, interventions that reduce IL-6 and increase insulin sensitivity and adiponectin could further decrease total WBC counts by reducing monocyte, lymphocyte, and basophil counts. However, aerobic exercise in post-menopausal women, even up to 150% of the recommended dose, does not appear to directly influence IL-6, adiponectin or monocyte, lymphocyte, or basophil counts.

The primary strength of the DREW study is that it was a tightly-controlled exercise intervention study in which the doses of exercise were strictly monitored in a laboratory setting leading to excellent adherence and retention rates. While this design reduces the real-world applicability, we can demonstrate the efficacy of different doses of exercise on health related outcomes, including WBC counts. A limitation of this study is that the sample of participants is restricted to sedentary, overweight/obese postmenopausal women with only moderately elevated WBC counts at baseline and we are therefore unable to evaluate the effect of exercise on WBC counts in men, premenopausal women, or women at greater risk of CVD as determined by supra-clinical levels of WBC counts. However, this study sample represents a significant proportion of U.S. women aged 45 to 75 years that are likely to benefit from exercise training. Last, we had only a limited number of cytokines that may be responsible for the change in total WBC and WBC subfraction counts in response to exercise. Future studies could investigate the anti-inflammatory effects of different exercise doses by measuring the balance between pro- and anti-inflammatory cytokines.

In conclusion, aerobic exercise training reduces total WBC and neutrophil counts, in a dose-dependent manner, in overweight/obese postmenopausal women after 6 months of exercise. The decreases in neutrophil counts were independent of changes in adiposity (BMI and waist circumference) and represent an approximate 4% reduction in CHD risk at the 2 highest doses of exercise, which includes the standard recommendation for general health (8 KKW). The dose-dependent decreases in total WBC and neutrophil counts were especially pronounced in women with systemic low grade inflammation.
